# Radio frequency heating and material processing using carbon susceptors†

**DOI:** 10.1039/d1na00217a

**Published:** 2021-07-30

**Authors:** Aniruddh Vashisth, Shegufta T. Upama, Muhammad Anas, Ju-Hyun Oh, Nutan Patil, Micah J. Green

**Affiliations:** Department of Mechanical Engineering, University of Washington Seattle WA USA vashisth@uw.edu; Artie McFerrin Department of Chemical Engineering, Texas A&M University College Station TX USA; Department of Materials Science & Engineering, Texas A&M University College Station TX USA

## Abstract

Carbon nanomaterials have been shown to rapidly evolve heat in response to electromagnetic fields. Initial studies focused on the use of microwaves, but more recently, it was discovered that carbon nanomaterial systems heat in response to electric fields in the radio frequency range (RF, 1–200 MHz). This is an exciting development because this range of radio frequencies is safe and versatile compared to microwaves. Additional RF susceptor materials include other carbonaceous materials such as carbon black, graphite, graphene oxide, laser-induced graphene, and carbon fibers. Such conductive fillers can be dispersed in matrices such as polymer or ceramics; these composites heat rapidly when stimulated by electromagnetic waves. These findings are valuable for materials processing, where volumetric and/or targeted heating are needed, such as curing composites, bonding multi-material surfaces, additive manufacturing, chemical reactions, actuation, and medical ablation. By changing the loading of these conductive RF susceptors in the embedding medium, material properties can be customized to achieve different heating rates, with possible other benefits in thermo-mechanical properties. Compared to traditional heating and processing methods, RF heating provides faster heating rates with lower infrastructure requirements and better energy efficiency; non-contact RF applicators or capacitors can be used for out-of-oven processing, allowing for distributed manufacturing.

## Abstract 2

This mini-review presents the science and applications of rapid heating of carbonaceous materials using radio frequency fields (1–200 MHz).

## Graphical abstract

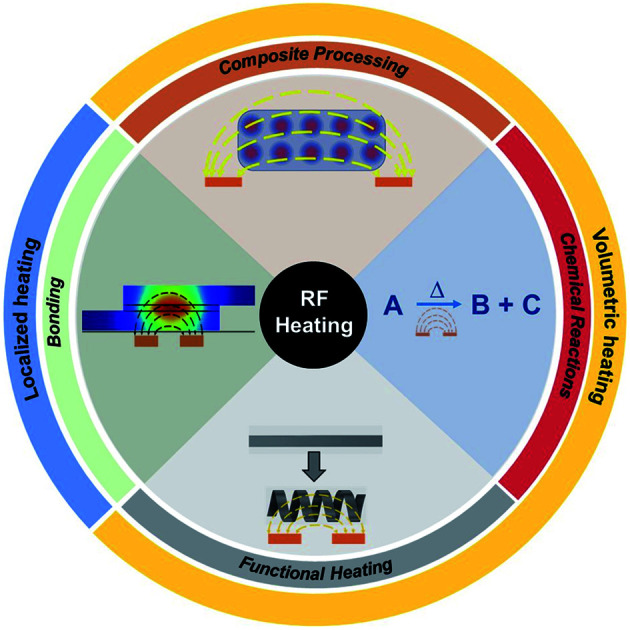

## Introduction

Heating technology has played a significant role in human history, from ancient Greeks using kilns for heating pottery^1^ to modern applications where aerospace and automotive composites are heated in large ovens.^2^ In recent years, methods of advanced manufacturing that require rapid heating or targeted heating of components have been developed.^3^ These include Joule or resistive heating,^4–7^ induction heating,^8^ vibrational heating,^9^ ultrasonic heating and welding,^10^ infrared heating,^11^ and electromagnetic heating.^12,13^ Specifically, electromagnetic heating is a rapidly emerging field that has several advantages including rapid, non-contact, non-invasive, and material-selective volumetric heating.

By coupling electromagnetic waves with conductive nanofillers such as carbon nanotubes (CNTs), rapid heating can be achieved. Recently, researchers first discovered that CNTs respond to microwave heating.^14,15^ The interaction of microwaves with CNTs is an exciting phenomenon that has been explored for applications ranging from material processing to medical treatments.^14^ Microwave-induced heating of CNTs has been used for various processing applications such as bonding,^16^ welding,^17^ curing thermosets,^18^ CNT functionalization,^19^ and heating CNT/silicone.^15^ Apart from CNTs, microwave-assisted processing of other carbon materials for synthesis, transformation, and thermal treatments have also been studied.^20,21^

Most of the prior work on heating carbon nanomaterials has been done using microwaves in GHz range.^14,15^ Recently, researchers found that RF fields between 1–200 MHz can be used to heat carbonaceous materials including CNTs,^22^ carbon black,^23^ graphene,^24^ and carbon fibers.^25^ The ability to use lower frequencies rather than microwaves for heating materials offers key benefits. These include greater penetration depths than microwaves, which allows for better heating in thicker materials. Radio frequency waves are capable of better selectivity; dielectric loss tangents of materials tend to be higher in the frequency range of microwaves, whereas at radio frequencies, more materials have comparatively low dielectric loss tangents, making them less susceptible to RF heating. This exciting discovery of RF heating has shown potential to reduce fabrication time and energy consumption for applications ranging from additive manufacturing^22^ to thermal reduction^24^ relative to status quo.

Here, we review the fundamental science and applications of RF heating in materials processing. This includes both the scientific underpinnings of the phenomena as well as the engineering design needs (volumetric heating and/or local heating) where RF heating can be valuable. This review focuses on research efforts that have utilized RF frequencies of 1–200 MHz for material processing. The safe use of RF heating has potential to unlock avenues in a wide range of applications such as out-of-oven distributed manufacturing, additive manufacturing, and chemical reaction engineering.

## Dielectric heating

**Fig. 1:**
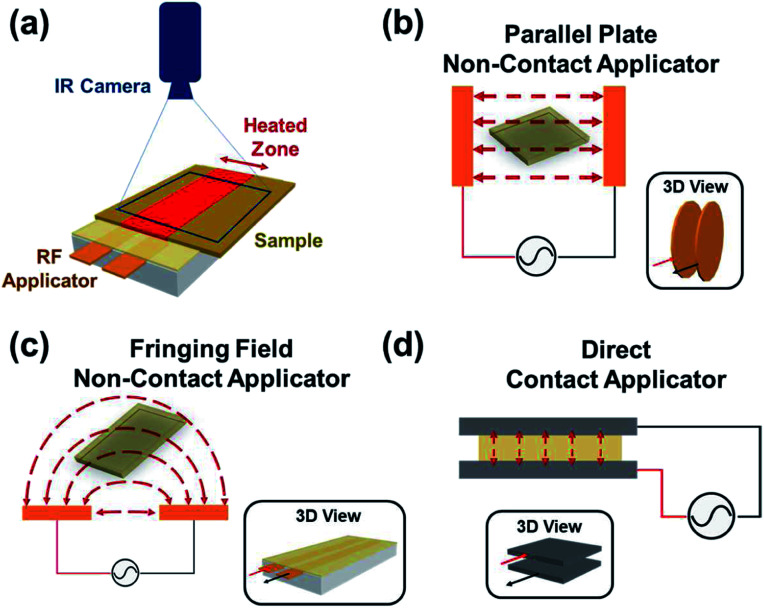
(a) Setup for RF heating of samples using a non-contact applicator, the thermal data is recorded by the FLIR camera. Variations of RF applications include two examples of non-contact applicators (b) parallel plate applicator and (c) fringing field applicator; and (d) direct-contact applicator where the metallic substrates are used as electrodes.^22^

A dielectric material contains either permanent or induced dipoles that polarize due to the rotation or finite displacement of charges in an electric field, resulting in heat generation.^26^ Radio frequencies can be used for dielectric heating of materials. To stimulate these materials with RF fields, an electric field applicator is required. Applicators may consist of two metallic electrodes, one of which is connected to a signal generator that carries the sinusoidal RF signal, and the other electrode is grounded. These applicators can couple RF fields with the sample in either a direct-contact or a non-contact manner, thus allowing for a variety of heating setups.^22^ A typical setup (Fig. 1a) consists of an applicator, a sample, and a temperature measurement device such as a forward-looking infrared (FLIR) thermal camera. For non-contact applicators, a time-varying electric field is generated between the ground and the RF signal electrode, resulting in dielectric heating of the sample. Non-contact RF applicators can be fabricated in a variety of geometries, including parallel plate applicators and fringing field applicators. Parallel plate applicators consist of two copper electrodes parallel to each other fixed on an insulating support bar (Fig. 1b); the fields generated between these two plates can be utilized to heat materials. A fringing-field applicator has two coplanar strips of electrodes sandwiched between a Teflon block with an insulating coating; in contrast to parallel plate applicators, the electrodes do not face each other, but rather lie on the same plane. This results in strong fields between the electrodes and some distance away curving around as shown in Fig. 1c. Fig. 1d shows an example of a direct-contact RF applicator where the electrodes are in contact with the sample that needs to be heated. Note that parallel plate non-contact applicators and metallic contact applicators have strong fields only between the electrodes; fringing field applicators have similar fields between the conductive electrodes and additional fields on the periphery, as shown in Fig. 1c. Given a frequency, the applicator geometry can be designed to minimize reflected power and maximize heating rates.

## Science of radio frequency heating

The mechanism for RF heating behavior can be examined at different length scales: both the molecular scale and bulk scale. Dielectric materials contain either permanent or induced dipoles; when placed between two electrodes, these materials act as capacitors allowing charge to be stored in them. The polarization of dielectrics arises from the finite displacement of charges or rotation of dipoles in an electric field. At the molecular level, polarization results in the physical rotation of molecular dipoles, causing radio frequency dielectric heating.^26^

In practice, the heating response of a material to an RF field is dependent on the bulk material properties and the electric field magnitude and frequency. A materials dielectric loss tangent (tan *δ* = *ε*′′/*ε*′) determines the ability of a material to be heated in the presence of electromagnetic fields. The dielectric loss tangent is comprised of two parameters: the dielectric constant or real permittivity (*ε*′), and the dielectric loss factor or imaginary permittivity (*ε*′′), and the complex permittivity is given by *ε* = *ε*′ − *iε*′′. The dielectric constant (*ε*′) determines the fraction of incident energy that is reflected and absorbed, while the dielectric loss factor (*ε*′′) measures the dissipation of electric energy in the form of heat within the material. For optimum electromagnetic energy coupling, a moderate value of *ε*′ should be combined with high values of *ε*′′ (resulting in high values of tan *δ*), to convert RF energy into thermal energy.^27,28^

**Fig. 2:**
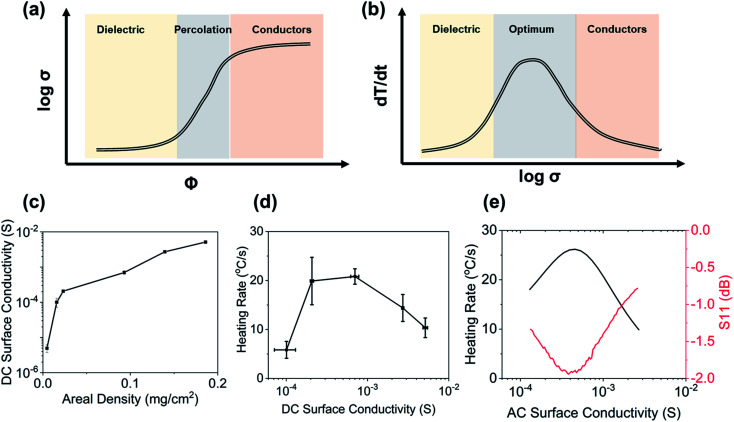
(a) General trends for conductivity as a function of conductive nanofiller loading; (b) instantaneous RF heating rates for nanocomposites with tailored conductivity; (c) experimental DC surface conductivity as a function of areal density for CNT films. (d) Experimental heating rate as a function of surface conductivity at 130 MHz and 2 W for CNT films. (e) Simulated heating rate and |*S*_11_ |as a function of surface conductivity at 130 MHz and 2 W for CNT films.^30^

For polymer composites, dielectric properties and conductivity can be tailored by addition of conductive particles; in doing so, conventional polymers with low conductivity transition from dielectrics to conductive materials (Fig. 2a). For polymer-based nanocomposites, this transition from a dielectric to a conductor happens when the conductive filler network percolates.^29^

However, the heating rate is a function of both material properties (bulk electrical conductivity) and the specimen geometry. For a specimen with a fixed geometry, maximum heating is achieved when the nanofillers form a percolated network within the embedded medium; before forming the percolated network, sufficient RF energy is not converted to heat, whereas at significantly higher conductivities, RF fields get reflected (Fig. 2b). Also, as the specimen's thickness increases, more input power is required to generate heating, as shown in eqn (1a). It should also be noted that penetration depth of the field is inversely proportional to the electrical conductivity of the bulk material (eqn (1b)); for highly conductive materials, the fields cannot penetrate deeper, thereby resulting in surface heating rather than volumetric heating.1a
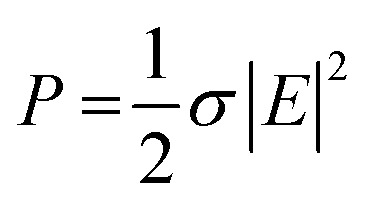
where *P* is power density in W m^−3^, *σ* is material conductivity in S m^−1^, and *E* is electric field strength in V m^−1^.1b
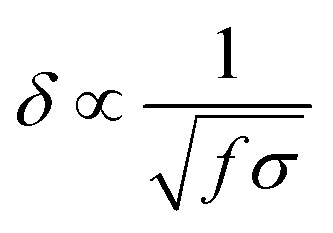
where *δ* is the penetration depth of the field and *f* is the frequency of the field.

The relationship between the heating rate and conductivity is non-monotonic; systems with very low conductivity (dielectric) and very high conductivity (conductor) do not produce a significant heating response (Fig. 2b). Low-conductivity samples don't respond to the field, but high-conductivity samples have a small *δ*. The precise shape of this graph is dependent on the sample size and the RF frequency.

Thus, by tailoring the conductivity of a material, a rapid RF response can be achieved. For instance, in a polymer-based composite, the electrical conductivity can be tailored by dispersing conductive carbonaceous nanofillers. It has been observed that by adding a very low weight percentage of CNTs, the polymer composite can be transitioned from a dielectric to a conductor.^29^ The concentration of conductive fillers should be close to the percolation threshold, resulting in the desired conductivity to achieve maximum heating in polymer composites.

Examples of this relationship have been also determined experimentally (Fig. 2c and d).^30^ As seen in Fig. 2c, an increase in surface density of CNTs results in a monotonic increase in the DC electrical surface conductivity of samples. The heating rate of these samples is non-monotonic with increasing conductivity (Fig. 2d); after a maximum is reached for instantaneous heating rates, the sample starts to reflect RF fields. Further, Fig. 2e shows the *S*_11_ (reflection coefficient) as a function of conductivity for a fixed frequency and power; the minima observed for the *S*_11_ for a specific conductivity confirm maximum absorption resulting in maximum heating rates.

## Benefits of dielectric heating

Conventional methods of manufacturing have low energy efficiencies and can require large production footprints. Usually, these methods require specimens to be placed in heated chambers; this leads to significant energy losses as the input power for heating is fixed and not governed by the specimen size and geometry. In specific applications, only a portion of an assembly needs to be targeted and heated, but conventional processing methods result in heating the whole assembly leading to distortions and residual stresses.

Dielectric heating using RF fields can circumvent a number of challenges faced by conventional methods of heating. Advantages include the following:

(1) Electromagnetic-thermal coupling: the energy from RF fields can drive heat generation or chemical reaction (curing of epoxy, reduction, *etc.*) in systems where the materials can be coupled strongly with electromagnetic waves leading to faster heating rates as compared to conventional methods. For example, using RF fields two plastic substrates were bonded to each other, reaching target temperatures (80 °C) within 45 seconds as compared to the state-of-the-art method that required ∼2 minutes using the IR lamps. In this case, the efficiency of RF heating was 6 times as that of IR heating.^23^

(2) Volumetric heating: bulk specimens embedded with RF susceptors can be heated inside-to-outside by RF fields; this method reduces the need for large ovens or furnaces to heat the specimens. Heating and partially/fully curing carbon fiber composites is an excellent example of RF rapid volumetric heating; heating composites in conventional ovens and autoclaves takes significant time (10–30 minutes) whereas using RF fields CF/epoxy systems can be heated as fast as 70 °C s^−1^ to attain curing temperatures within 2 minutes of the cure-cycle.^25^

(3) Targeted heating: in assemblies that require heating on specific locations, RF fields can provide targeted heating for applications such as bonding. This method bypasses the need to heat the whole assembly, potentially leading to residual thermal stresses in the assembly once it cools down. This has shown application for improving inter-layer adhesion in 3D printed structures, with improvements in weld fracture strength by 275%.^31^

(4) Non-contact processing: RF energy can be introduced into the target material remotely; no direct contact is required between the energy source and the reacting system.

(5) Tailored response: as shown in Fig. 2, the heating rates for a set of RF frequency and input power are dependent on the material's bulk conductivity; for polymer composites, this can be tuned by varying the concentration of carbonaceous nano- and micro-fillers to achieve desired heating rates. For example, varying MXene concentration in polymer composite films from 1 wt% to 25 wt%, heating rates can be increased from ∼0.1 °C s^−1^ to ∼30 °C s^−1^.^32^ Further, conductive coatings or polymers can be patterned to achieve different heating rates in a single specimen. The heating rates can also be tailored by changing RF frequency and input power; for example, heating rates vary between 0–19 °C s^−1^ for multiple frequencies between 1–200 MHz at 2 W of input power.^30^ Also heating rates of carbon fiber composites jump from 10 °C s^−1^ to 70 °C s^−1^ when input power is increased from 5 W to 25 W keeping a fixed frequency of 66 MHz.^25^

(6) Low infrastructure: in terms of instrument footprint, dielectric heating has reduced production floor-space requirements. For example, typical composite autoclaves are cylindrical in shape and the smallest of these structures have 4′ inner diameters and are 6′ long plus the control systems; whereas RF heating systems consist of an RF amplifier and signal generator, the size of the RF applicator can be tailored according to requirements.

In addition, RF heating can be advantageous on a society-wide scale for use in the manufacturing and processing industries. RF heating of nanomaterials can enable distributed manufacturing and usage, rather than centralized manufacturing and distributed usage; this could lead to a massive saving in transportation and reduce related greenhouse gas emissions. This green manufacturing effort has the potential to become part of a larger manufacturing industry-wide thrust toward the use of electricity, rather than steam utilities or fossil fuel combustion, to generate heat for manufacturing. Also, RF heating requires reduced floor space, resulting in low infrastructure cost and capital investment; the method allows for out-of-oven rapid processing, thereby mitigating the need for installation of large ovens or autoclaves for fabrication.

Carbonaceous materials are particularly advantageous as RF susceptors because they are already used in a range of industrial applications. Examples include conventional carbonaceous materials such as graphitic fillers in car tires and carbon fiber-reinforced airplane wings.^33,34^ In addition, carbon-based nanomaterials have become more widely available because of improvements in bulk synthesis.

## Applications

**Fig. 3:**
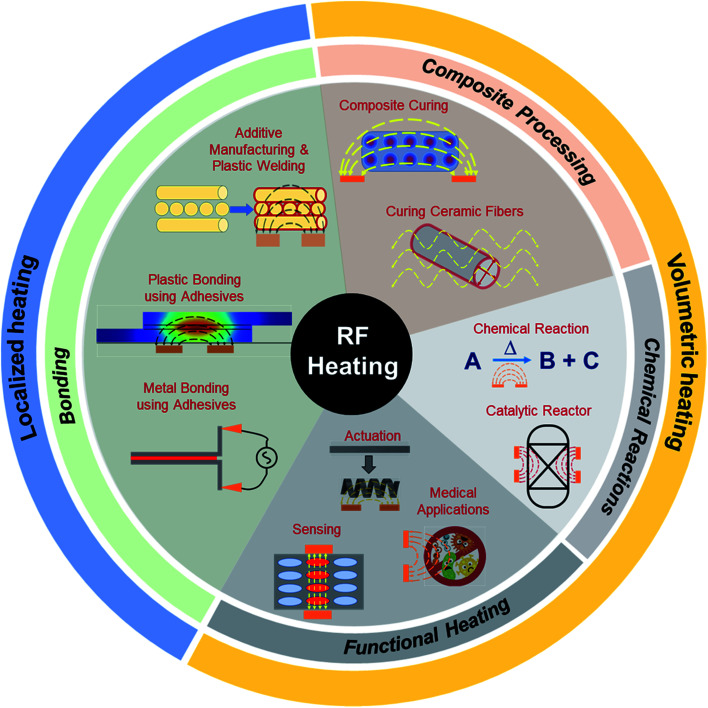
Schematic of RF heating applications for volumetric and localized heating, including bonding, composite processing, chemical reactions, and functional heating.

The application of RF heating can be broadly classified into two sub-categories: (i) volumetric heating, where heating is desired in bulk specimens for purposes of curing or initiating and sustaining reactions; and (ii) targeted heating, where only a section of the specimen needs to be heated, such as heating an interface for bonding or heating sections of a composite for actuation. Fig. 3 shows a wide range of applications of RF heating, ranging from volumetric heating for composite processing, initiating reactions and functional heating, and targeted heating applications for additive manufacturing and bonding.

### Volumetric heating

**Fig. 4:**
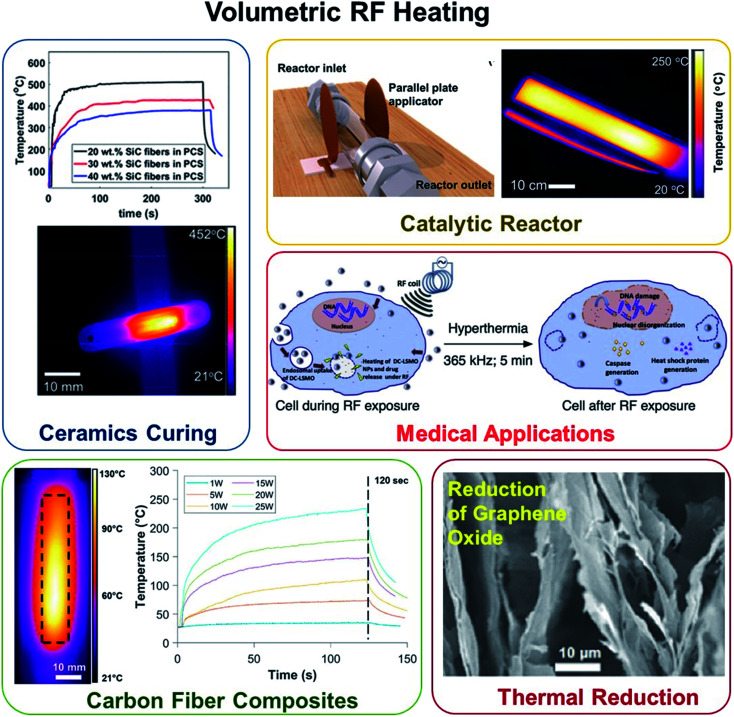
Volumetric RF heating has successfully shown application in (counterclockwise, from top-right) ceramics and carbon fiber composite fabrication, catalytic reactors, thermally driven chemical reactions, and medical applications.^25,35–37^

Heat generated within a structure that gets distributed throughout the volume is termed volumetric heating. This is achieved *via* RF susceptors embedded in the sample that gets heated when stimulated by RF fields, which eventually heat the overall structure. Volumetric RF heating has applications in composite processing, chemical reactions, and functional heating (Fig. 4).^25,35–37^

RF fields have successfully shown composite processing capabilities such as curing of carbon fiber (CF) reinforced thermosetting composites and out-of-oven ceramic manufacturing using multiple susceptors. Thermosetting prepregs are fabricated continuously by using energy-expensive methods such as large convection ovens, or infra-red lamps.^34^ Vashisth *et al.*^25^ demonstrated that unidirectional carbon fiber-epoxy prepregs can be heated up to 70 °C s^−1^ using only 25 W of input RF power *via* fringing-field applicators. The method was also tested for continuously heating prepregs by scanning them over RF applicators using varying translating speeds. The study showcased that RF fields can be used for tailored processing of CF thermosets, ranging from fabrication of prepregs to potentially out-of-oven processing of composite structures. For enabling out-of-oven ceramic manufacturing, Patil *et al.* studied two RF susceptors: multiwalled carbon nanotubes (MWCNTs)^36^ and silicon carbide (SiC) fibers.^37^ The addition of multiwalled carbon nanotubes allowed for rapid RF curing of preceramic polymers bypassing the commonly observed viscosity drop at the melting point. This technique for curing preceramic polymers can be engineered for rapid 3D printing of silicon carbides and fiber manufacturing. Further, the presence of turbostratic carbon on the surface of Hi-Nicalon SiC fibers results in the significant heating of these fibers under RF fields. RF heating response of commercially available Hi-Nicalon SiC fibers was used for fabrication of SiC/SiC ceramic matrix composites.

Volumetric heating using RF fields has shown promise in surface reactions such as reduction of graphene oxide (GO), and also in low infrastructure chemical reactors. Debnath *et al.*^24^ showed that RF heating *via* non-contact fringing-field applicator can be successfully used for rapidly heating and reducing GO, both in its neat form and inside polyvinyl alcohol (PVA) polymer matrix. Instantaneous heating rates for GO and GO-PVA composites were found to be 10.9 °C s^−1^ and 1.5 °C s^−1^ respectively, at 5 W input power. RF-reduced GO and GO-PVA samples have shown conductivities of 10^2^ S m^−1^ and 10^−1^ S m^−1^, respectively. Further, Patil and Mishra *et al.*^38^ demonstrated a proof-of-concept for RF powered catalytic reactors that leverage RF responsive nanomaterials such as CNTs and SiC fibers. This is a potential breakthrough over conventional catalytic reactors in that it enables safe, sustainable, on-site, and on-demand distributed chemical production in the absence of traditional manufacturing infrastructure.

RF energy also enables functional heating for actuation, sensing behavior, and decontamination of medical devices. Oh *et al.*^39^ demonstrated that site-specific thermal actuation could be achieved by using RF heating of carbon nanomaterials. Here, the driving mechanism is the difference in coefficients of thermal expansion that lead to bending in layered structures. Interestingly, site-specific actuation can be realized in these geometries by simply manipulating the field frequency; such actuators have potential applications in soft robotics, and micro-manipulation systems. Another application entails the quick assessment of circuit quality by examining their thermal patterns in response to RF fields.^40^ This new method screens CNT circuits ten times faster than conventional methods, identifying faulty circuits more reliably. Non-contact heating of melt electrospun polymer fibers embedded with CNTs can be achieved by using RF fields for applications such as plastic electric heaters, hyperthermia treatment, and heat-generating textiles.^41^ For medical applications, RF plasma can be used for sterilization of surgical and dental devices to eradicate cellulose and bacterial spores,^42,43^ and other contaminants;^44^ furthermore, dental devices can also be sterilized by RF fields.^45^ Additionally, RF fields can be used for hyperthermia and drug release,^46–48^ and non-invasive body fat reduction.^49,50^ Other notable applications include soil decontamination and drying of brickwork,^51,52^ adsorptive catalytic off-gas,^51^ oil recovery,^53^ sterilization of vacuum-packaged food,^54,55^ and non-chemical pest disinfestation.^56^

### Localized or targeted heating

**Fig. 5:**
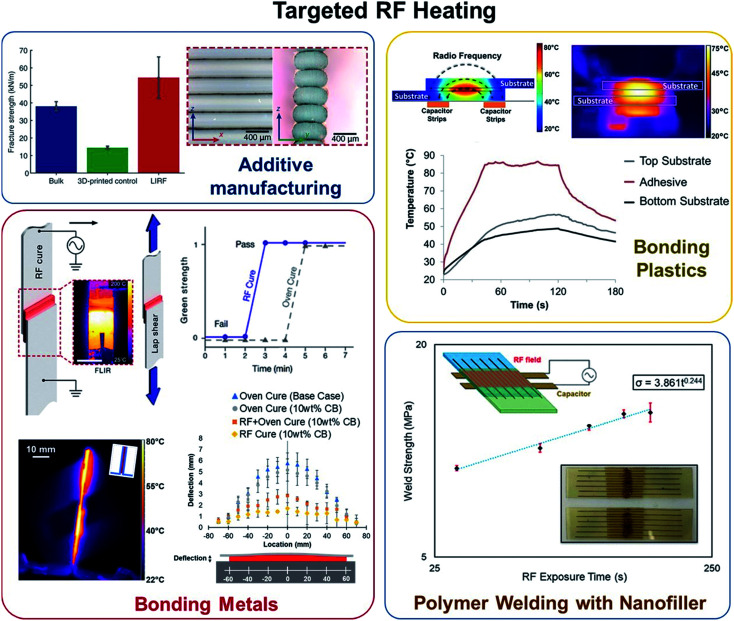
Successful application of targeted RF heating has been shown in (clockwise, from top-left) additive manufacturing for improved fracture structure, bonding plastics with locally heated adhesives, plastic welding, and metal–metal bonding using adhesives.^22,23,31,57^

Various methods have previously been developed for localized or targeted heating. The key concept is to deliver energy only to a targeted area or volume in a large assembly, to be dissipated as heat for melting or curing polymers (Fig. 5).

Targeted heating for bonding using RF fields has shown faster processing time as compared to conventional methods because of the heat-transfer advantages of directly heating the bondline.^22^ Different strategies are used to bond non-conducting substrates such as polymers and conductive substrates such as metallic structures. For bonding plastics, Gruener *et al.*^23^ showed that non-contact fringing field RF applicators could successfully heat and cure epoxy loaded with carbon black nanofillers; this method of RF heating was found to be six times more efficient than IR heating for bonding polypropylene plastic substrates. Thermoplastics can be welded together without using adhesives; this can be achieved by introducing a thin layer or patterns of RF responsive materials on the plastic substrates.^57^ Gerringer *et al.*^57^ showed that Laser-Induced Graphene (LIG) can be fabricated on polymer surface by using CO_2_ lasers; exposure of LIG to RF fields results in the rapid heating of LIG (up to 126 °C s^−1^) and welding the polymer–polymer interface. To bond metallic substrates, the metal components can be used as electrodes to heat and cure the adhesive sandwiched between the two conductive plates.^58^ The study found bonding two dissimilar metals using RF fields results in a significant reduction in distortions due to CTE mismatch as compared to conventional oven cured specimens. Targeted RF heating can be used in additive manufacturing for remelting the polymer at the interlayer and improving out-of-plane strength and fracture strength; Sweeney *et al.*^31^ showed that 3D printed parts with CNT coated polymer filaments and post-treated by RF fields can successfully show 275% improvement in fracture strength as compared to conventional 3D printed parts.

Table S1† lists the behavior of various carbonaceous materials to RF fields used for varying applications. It should be noted that the RF heating response of different carbon susceptors is dependent on various factors such as geometry of RF applicators, input power and frequency, embedding medium, volume of sample that is being heated, and distance from the RF applicator. Nanomaterials are usually chosen due to their multifunctionality; for example LIG is beneficial for polymer welding since it is easy to fabricate on polymer surfaces^57^ whereas CNTs provide excellent heating at low concentrations in adhesives but also improve mechanical properties.^22^ Anas *et al.*^30^ provide a detailed overview of RF heating science (through experiments and simulations) generalized across a wide range of nanomaterials based on dielectric properties.

## Summary and outlook

In this mini-review, the growing applications of radio frequency (RF) heating have been highlighted. The non-contact processing with rapid heating rates can be exploited by various industries ranging from automotive to chemical synthesis to sterilization of medical devices.^23,24,45^ Most of these advances have been made in the last 5–7 years and show immense potential for revolutionizing the manufacturing and processing industry. This novel method of heating using RF fields has seen substantial intellectual property development.^59–62^ Some of these concepts have seen translation from academic labs to industry.^5^ RF heating has demonstrated potential for rapid manufacturing applications for heating bulk materials or targeted parts of the specimens through volumetric or targeted heating.^22,24,25^ Major questions surrounding this phenomena remain open. These include but are not limited to: new and novel materials that can be used as RF susceptors, understanding the interplay between nano-scale and macro-scale heating in nanocomposites, and shielding nearby electronics from RF interactions.

Overall, rapid heating for material processing using RF fields specifically is of great interest as this can decrease the processing time scales significantly, which would imply a reduced energy consumption. RF heating and processing can be used for distributed manufacturing, thereby significantly improving the lead times on material fabrication (especially in remote locations of the world); the final product achieved by RF-assisted processing will be more economically competitive than one obtained by traditional techniques.

## Conflicts of interest

There are no conflicts to declare.

## Supplementary Material

NA-003-D1NA00217A-s001
