# Supplementary material for: Radio frequency heating and material processing using carbon susceptors
Source: Nanoscale Adv. 2021 Jul 30;3(18):5255–64. doi: 10.1039/d1na00217a (PMC9419054; doi:10.1039/d1na00217a)
Supplement: NA-003-D1NA00217A-s001 [file NA-003-D1NA00217A-s001.pdf]

Electronic Supplementary Material (ESI) for Nanoscale Advances.
This journal is © The Royal Society of Chemistry 2021

Supporting Information

Radio Frequency Heating and Material Processing Using Carbon Susceptors

Aniruddh Vashisth[1]*, Shegufta T. Upama[2,3], Muhammad Anas[2], Ju-Hyun Oh[2], Nutan Patil[2], Micah J. Green[2,3]

[1]Department of Mechanical Engineering, University of Washington, Seattle, WA, USA

[2]Artie McFerrin Department of Chemical Engineering, Texas A&M University, College Station, TX, USA

[3]Department of Materials Science & Engineering, Texas A&M University, College Station, TX, USA

*corresponding author: vashisth@uw.edu

Table S1: Reported heating response of carbonaceous materials on exposure to RF fields and its applications.

RF Susceptor	Embedding Medium	Input power (W)/ Frequency (MHz)	Max reported dT/dt (°C/s) or Max T (°C) @ SS	Application [Reference]
MWCNT (0.1-10wt%)	Polylactic acid	10 W /1-200 MHz (Fringing Field)	16 °C/s	Bonding [1]
s-SWCNT	DOC	1 W/60–70 MHz	25.6 °C/s	Film purity [2]
m-SWCNT	DOC		10.4 °C/s	
m-SWCNT	SDS		1.51 °C/s	
MWCNT (3 wt%)	Polycarbosilane	100 W/76–78 MHz	600 °C (SS)	Ceramic Precursor curing [3]
MWCNT (0.1 wt%)	Polycaprolactone	50 W/110 MHz	2 °C/s	Heating Fibers [4]
LIG	None	10 W/100-120 MHz	21–122 °C/s	Welding Polymers [5]
SiC fiber	N/A	30 W/100 MHz	400 °C (SS)	Catalytic Reactor [6]
GO	None	5 W/107-108 MHz	10.9 °C/s	GO reduction [7]
GO (15 wt%)	Polyvinyl Alcohol		1.5 °C/s	
Carbon Tape	None	25 W/101 MHz	208 °C (SS)	Thermal actuator [8]
LIG		63 W/101 MHz	202 °C (SS)	
Carbon Fibers	Epoxy	25 W/ 66 MHz	70 °C/s	Curing CF composites [9]
CB	Structural Adhesive	10 W/ 138 MHz	1 °C/s	Adhesive bonding of metals [10]
CB	Structural Adhesive	100 W/114 MHz	~3.5 °C/s	Adhesive bonding of plastics [11]
Graphene	None	5 W/ 70–130 MHz	Up to 25 °C/s	Identify type of graphene [12]
PAN films	None	30 W/ 106 MHz	280 °C (SS)	Crosslinking [13]

SS: Steady state; dT/dt: (change in Temperature)/(change in time); MWCNTs: Multi-wall carbon nanotubes; m- or s-SWCNTs: metallic or semiconducting Single-wall carbon nanotubes; GO: Graphene oxide; LIG: Laser induced graphene; SiC: Silicon carbide; CB: Carbon black; SDC: Sodium dodecyl sulfate; DOC: Sodium deoxycholate

References

(1) Sweeney, C. B.; Moran, A. G.; Gruener, J. T.; Strasser, A. M.; Pospisil, M. J.; Saed, M. A.; Green, M. J. Radio Frequency Heating of Carbon Nanotube Composite Materials. *Acs Appl Mater Inter* **2018**, *10*, 27252-27259.

(2) Anas, M.; Zhao, Y.; Saed, M. A.; Ziegler, K. J.; Green, M. J. Radio frequency heating of metallic and semiconducting single-walled carbon nanotubes. *Nanoscale* **2019**, *11*, 9617-9625.

(3) Patil, N.; Camacho, A. C.; Mishra, N. K.; Singhla, P.; Sweeney, C. B.; Saed, M. A.; Radovic, M.; Green, M. J. Radio Frequency and Microwave Heating of Preceramic Polymer Nanocomposites with Applications in Mold-Free Processing. *Adv Eng Mater* **2019**, *21*, 1900276.

(4) Morikawa, K.; Vashisth, A.; Bansala, T.; Green, M. J.; Naraghi, M. Wire Melt Electrospun Polymer Nanocomposite Fibers as Radio Frequency Responsive Heaters. *ACS Applied Polymer Materials* **2019**, *1*, 2751-2759.

(5) Gerringer, J. C.; Moran, A. G.; Habib, T.; Pospisil, M. J.; Oh, J. H.; Teipel, B. R.; Green, M. J. Radio Frequency Heating of Laser-Induced Graphene on Polymer Surfaces for Rapid Welding. *ACS Applied Nano Materials* **2019**, *2*, 7032-7042.

(6) Patil, N.; Mishra, N. K.; Saed, M. A.; Green, M. J.; Wilhite, B. A. Radio Frequency Driven Heating of Catalytic Reactors for Portable Green Chemistry. *Advanced Sustainable Systems* **2020**, *n/a*, 2000095.

(7) Debnath, D.; Zhao, X. F.; Anas, M.; Kulhanek, D. L.; Oh, J. H.; Green, M. J. Radio frequency heating and reduction of Graphene Oxide and Graphene Oxide - Polyvinyl Alcohol Composites. *Carbon* **2020**, *169*, 475-481.

(8) Oh, J. H.; Anas, M.; Barnes, E.; Moores, L. C.; Green, M. J. Site-Specific Selective Bending of Actuators using Radio Frequency Heating. *Adv Eng Mater* **2021**, *n/a*, 2000873.

(9) Vashisth, A.; Healey, R. E.; Pospisil, M. J.; Oh, J. H.; Green, M. J. Continuous processing of pre-pregs using radio frequency heating. *Compos Sci Technol* **2020**, *195*, 108211.

(10) Vashisth, A., Auvil, T.J., Sophiea, D., Mastroianni, S.E., Green, M.J. Using Radio Frequency Fields for Local Heating and Curing of Adhesive for Bonding Metals. *(Submitted)* **2021**.

(11) Gruener, J. T.; Vashisth, A.; Pospisil, M. J.; Camacho, A. C.; Oh, J.-H.; Sophiea, D.; Mastroianni, S. E.; Auvil, T. J.; Green, M. J. Local heating and curing of carbon nanocomposite adhesives using radio frequencies. *Journal of Manufacturing Processes* **2020**, *58*, 436-442.

(12) Mason, M. J.; Coleman, B. J.; Saha, S.; Mustafa, M. M.; Green, M. J. Graphene signatures: Identifying graphite and graphene grades via radio frequency heating. *Carbon* **2021**, *182*, 564-570.

(13) Patil, N.; Oh, J. H.; Khatri, S.; Saed, M. A.; Naraghi, M.; Green, M. J. Radio Frequency Heating Response of Polyacrylonitrile (PAN) Films and Nanofiber Mats. *ACS Applied Polymer Materials* **2021**, *3*, 3125-3130.
